# Label-free and amplification-free viral RNA quantification from primate biofluids using a trapping-assisted optofluidic nanopore platform

**DOI:** 10.1073/pnas.2400203121

**Published:** 2024-04-10

**Authors:** Mohammad Julker Neyen Sampad, S. M. Saiduzzaman, Zach J. Walker, Tanner N. Wells, Jesse X. Wayment, Ephraim M. Ong, Stephanie D. Mdaki, Manasi A. Tamhankar, Thomas D. Yuzvinsky, Jean L. Patterson, Aaron R. Hawkins, Holger Schmidt

**Affiliations:** ^a^School of Engineering, University of California, Santa Cruz, CA 95064; ^b^Electrical and Computer Engineering Department, Brigham Young University, Provo, UT 84602; ^c^Texas Biomedical Research Institute, San Antonio, TX 78227

**Keywords:** solid-state nanopore, single-molecule detection, viral RNA, label-free, amplification-free

## Abstract

Viral outbreaks can cause widespread disruption, creating the need for diagnostic tools that provide high performance and sample versatility at the point of use with moderate complexity. Current gold standards such as PCR and rapid antigen tests fall short in one or more of these aspects. Here, we report a label-free and amplification-free nanopore sensor platform that overcomes these challenges via direct detection and quantification of viral RNA in clinical samples from a variety of biological fluids. The assay uses an optofluidic chip that combines optical waveguides with a fluidic channel and integrates a solid-state nanopore for sensing of individual biomolecules upon translocation through the pore. High specificity and low limit of detection are ensured by capturing RNA targets on microbeads and collecting them by optical trapping at the nanopore location where targets are released and rapidly detected. We use this device for longitudinal studies of the viral load progression for Zika and Severe Acute Respiratory Syndrome Coronavirus-2 (SARS-CoV-2) infections in marmoset and baboon animal models, respectively. The up to million-fold trapping-based target concentration enhancement enables amplification-free RNA quantification across the clinically relevant concentration range down to the assay limit of RT-qPCR as well as cases in which PCR failed. The assay operates across all relevant biofluids, including semen, urine, and whole blood for Zika and nasopharyngeal and throat swab, rectal swab, and bronchoalveolar lavage for SARS-CoV-2. The versatility, performance, simplicity, and potential for full microfluidic integration of the amplification-free nanopore assay points toward a unique approach to molecular diagnostics for nucleic acids, proteins, and other targets.

## Significance

The high infectivity and rapid transmission of viral diseases make them prone to causing pandemics, as evidenced by several examples in the past two decades. We have developed an integrated optofluidic nanopore platform for label-free and amplification-free quantification of viral RNA loads across the course of an infection from a variety of biological sample fluids. An attomolar limit of detection is enabled by optical trapping–assisted enhancement of the target concentration at the nanopore by many orders of magnitude. Viral loads from six different biofluid types from two different primate models for Zika and SARS-CoV-2 infection are quantified with the same performance as RT-qPCR, but at dramatically reduced complexity, pointing the way toward a unique class of universal biomarker diagnostics.

Infectious diseases continue to be a strain on society due to numerous factors, including high rates of infectivity, rapid transmission routes, variable incubation periods before showing symptoms, frequent genome mutations, and lack of vaccines and treatments (1–3). Over the past decades, the world has witnessed multiple viral pandemics such as the Swine flu pandemic in 2009, the 2013 to 2016 Ebola pandemic, the 2015 to 2016 Zika pandemic, and the COVID-19 pandemic, causing huge loss of life as well as major economic and societal disruptions (4–7). As preventive measures such as vaccinations and antiviral medicine are often either limited or unavailable, it is essential to develop simple, low-cost, and low-complexity point-of-care (POC) diagnostic technologies with high sensitivity, speed, and accuracy (8, 9). Most of the currently available viral detection methods target specific viral nucleic acids and protein biomarkers (10, 11). While rapid and inexpensive antigen (protein) tests such as enzyme-linked immunosorbent assay are suitable for POC applications, they suffer from poor sensitivities and reliabilities when the biomarker concentration is low (12). As a result, qPCR methods that amplify nucleic acid targets remain the gold standard for viral diagnostics due to their high specificity and sensitivity (13). However, PCR is complex and requires expensive reagents, central laboratory infrastructure, and well-trained personnel, making it ill-suited for use in low-resource environments (14). Specifically, assay complexity manifests in the need for thermal cycling, a RT step for RNA virus detection, problems with the amplification process, and the need for a standard calibration curve for quantitative viral load analysis (15, 16). Emerging technologies such as RT Loop-Mediated Isothermal Amplification offer some simplification by avoiding thermocycling and using a simple colorimetric readout process. However, this technique still utilizes the complex reverse transcription–assisted nucleic acid amplification-based method (17, 18). Recently, integrated single-molecule analysis using fluorescence detection of individual biomarkers in microfluidic channels has enabled direct detection of viral nucleic acids and proteins at clinically relevant concentrations (19, 20). However, until now, no approach that combines the low-complexity of label- and amplification-free sensing with a low limit of detection (LOD) and wide dynamic range in a portable lab-on-chip format has been demonstrated in clinical samples.

Direct single-molecule detection with integrated electrical nanopore sensors can meet this need. Nanopore detection is based on changes in ionic current across a membrane with a nanoscopic opening as a particle passes (translocates) through this nanopore (21). This principle now forms the basis of commercial next-generation DNA sequencing technology (22) but has also been used to detect other biomolecules, including DNA (23–25), RNA (26, 27), proteins (28, 29), whole virus (30, 31), and small molecules (32, 33). Thus, nanopores offer outstanding (single-molecule) sensitivity and great simplicity due to their label-free transduction mechanism. However, nanopore sensors face two main challenges that have so far precluded their more widespread use as universal particle sensors and as diagnostic tools in particular. The first issue is the lack of specificity in identifying the translocating particle which creates ambiguity when working with a complex biofluid. Existing remedies such as nanopore surface modification with selective molecular receptors (34–36), target modification with custom-made DNA aptamers (37–39) or nanoparticles (40), and surface-functionalized magnetic bead-based target capturing and filtration (41) significantly increase complexity, thus negating some of the main benefits of the nanopore approach. The second limitation is low throughput due to a limited target detection rate. The electric field that promotes target translocation through the nanopore is confined to a few micrometers outside the pore. As a result, particle transport to the high-field region in the vicinity of the pore has traditionally been diffusion limited. At clinically relevant biomarker concentrations in the femtomolar and attomolar range, this leads to impractically low detection rates. Several methods to facilitate target delivery to the pore have been pursued, including modification of internal charge in protein pores (42), establishing pressure gradients (43), salt gradient–based electric field enhancement in the analyte side (44), plasmonic heating–based thermophoretic target capture (45), dielectrophoretic trapping of nucleic acids with metal-coated nanopore pipette (46), and isotachophoresis-based delivery (47). However, these approaches also add complexity or are incompatible with a POC instrument, and so the full realization of the promise of nanopore sensing as the basis of a label-free and amplification-free diagnostic has remained unfulfilled.

We have recently introduced an optofluidic approach to nanopore detection that solves these challenges to create a new, powerful method for nanopore-based biomarker analysis (48). It is based on solid-phase extraction of nucleic acids onto functionalized microbeads to ensure assay specificity. These microbeads are then optically trapped within the electrical capture radius of the nanopore where the targets can be released and translocated through the nanopore in rapid succession. The potential of this trapping-assisted capture rate enhancement (TACRE) was demonstrated with synthetic oligomers (48), and SARS-CoV-2 RNAs spiked into nasal swab samples (49).

Here, we report the validation of this concept as a clinical diagnostic tool with the performance of RT-qPCR, but with dramatically reduced complexity. Two longitudinal studies of viral infection in primate animal models—marmosets for Zika virus and baboons for SARS-CoV-2—were conducted. Six different biofluids [Zika: semen, urine, and whole blood; SARS-CoV-2: nasopharyngeal and throat swab (NPT), rectal (REC) swab, and bronchoalveolar lavage (BAL)] were monitored over 4 wk and analyzed both with RT-qPCR and with the optofluidic nanopore chip. First, we demonstrate that all sample types can be detected on the nanopore chip with a detection limit of 10 aM and a dynamic range of five orders of magnitude, allowing us to follow the course of infection and compare viral load magnitudes and dynamics of different biofluids. Second, we find that the nanopore sensor produces qualitative and quantitative agreement with all PCR-positive samples and was able to deliver a viral load reading for multiple samples that did not produce a PCR result, likely due to the complexity of that method. These results suggest that optofluidic nanopore sensing can form the basis of a unique paradigm for clinical biomarker diagnostics as well as a research tool for animal model development and other applications.

## Results

### Optical Trapping–Assisted Nanopore Capture Rate Enhancement Experimental Setup and Realization.

**Fig. 1.**
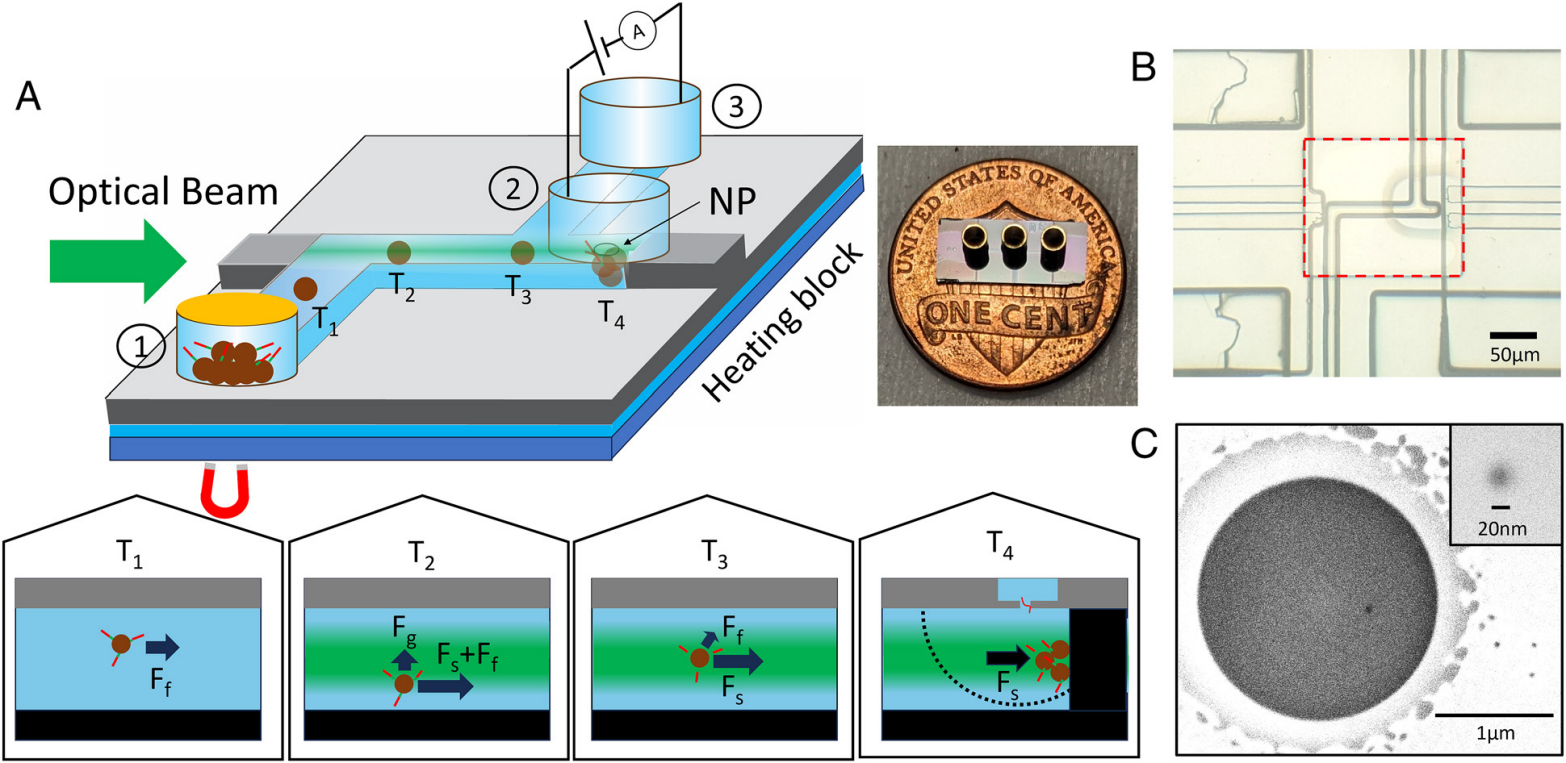
(*A*) Optical trapping–assisted nanopore capture rate enhancement chip. A microfluidic channel (blue) is intersected by a solid-core waveguide (gray) for optical microbead trapping inside a protrusion region. Magnetic microbeads are introduced to the channel by fluidic reservoirs and a drop of mineral oil creates fluidic pressure–based flow due to different liquid evaporation rates between the inlet and outlet reservoirs. The timepoints (T_1_–T_4_) illustrate the different forces experienced by the magnetic beads as they progress through the channel (see main text for discussion). Target detection takes place at T_4_ where the beads are trapped against the channel wall, very close to the nanopore (NP). The black dotted line indicates the electrical nanopore capture radius. A nanopore reservoir is attached for nanopore ionic current sensing. The *Inset* shows a photograph of the chip. (*B*) Optical microscope image of the experimental device. A red dashed rectangle shows a 300 nm thin membrane region for facilitating nanopore fabrication. (*C*) Scanning electron microscope (SEM) image of a 2 μm diameter circular microwell drilled by FIB to further thin the 300 nm SiO_2_ membrane. The *Inset* shows a zoomed-in photo of a FIB drilled 20 nm nanopore.

Fig. 1*A* shows a schematic view of the nanopore-integrated optofluidic platform and the experimental setup. Details of the optofluidic chip fabrication and solid-state nanopore integration are provided in the *Materials and Methods* section. Briefly, a 10 μm × 10 μm cross-section microfluidic channel (shown in blue) on a 12 mm × 5 mm silicon chip (Fig. 1 *A*, *Inset*) is defined by selective etching and an SiO_2_ deposition process. At the center of the device, the microfluidic channel intersects with a 10 μm × 6 μm solid-core (SC) waveguide (shown in gray) and creates a 100 μm long horizontal optofluidic particle manipulation region. The end of the horizontal optofluidic section is extended by 20 μm to create a low-flow protrusion cavity for isolating particles away from the fluid flow in the main channel (50). A 300 nm thin SiO_2_ membrane is selectively formed around the central optofluidic region to encapsulate the channel as well as to provide a thin membrane for easy nanopore integration (Fig. 1*B*). A complete longitudinal cross-section of the chip can be found in *SI Appendix*, Fig. S1*A*. A 20 nm nanopore is milled into the oxide membrane above the protrusion with a focused ion beam (FIB) (Fig. 1*C*). Three metallic reservoirs are attached with wax to provide fluidic and electrical access to openings in the microfluidic channel. Ag/Ag electrodes are used to create an electric potential difference between the nanopore (#2) and outlet (#3) reservoirs and create a ~2,200 µm^3^ target capture volume in the vicinity of the nanopore (for a visualization of this volume see *SI Appendix*, Fig. S1*B*). A patch-clamp current amplifier (Axopatch 200B, Molecular Devices) is connected to the electrodes to measure the ionic current signal across the nanopore.

The optical TACRE process is achieved by first immobilizing target nucleic acids from a biofluid on magnetic microbeads and then optically trapping the microbeads within the capture radius of the nanopore. Previously, we have demonstrated an implementation of this technique where electrophoretically driven particles were optically trapped against the channel wall directly underneath the nanopore (49), but the beads remained exposed to fluid flow forces while being trapped which increased the likelihood of inadvertently washing away targets after release from the beads. The present design isolates the beads from fluid flow by pushing them into the protrusion. This enabled us to improve reproducibility and further lower the LOD of the assay. Particles were moved through the channel via hydrostatic pressure from different fluid heights in the reservoirs. Detailed bead delivery rate analysis for this particle delivery method is available in *SI Appendix*, Fig. S2. Furthermore, the magnetic beads are preconcentrated inside the inlet reservoir using a brief magnetic pull-down to improve the bead delivery rate and keep the assay time in an optimal range (49). For optofluidic bead manipulation, a 532 nm laser is coupled to the solid-core optical waveguide by a single-mode fiber.

The process of delivering the target-carrying beads to the nanopore can be divided into four distinct timepoints (T_1_–T_4_, see Fig. 1*A*), during which various combinations of forces act upon a bead as it moves through the fluidic channel. Right after entering the fluidic channel (timepoint T_1_), the bead is propelled by the fluid flow-induced force F_f_.

Starting at the waveguide-channel intersection, an optical force composed of gradient (F_g_) and scattering force (F_s_) acts on the microbead (timepoint T_2_). The SC waveguide is aligned with the microfluidic channel so that the optical mode profile of the SC waveguide is centered in the channel cross-section (50). Thus, the gradient force pulls the bead to the center of the channel. The scattering force acts in the direction of light propagation, pushing the particle along the channel in the same direction as the fluid flow. At the junction where the protrusion is located (timepoint T_3_), the bead is pushed into the protrusion if the scattering force exceeds the fluid flow force. This approach has been shown to deliver particles into the protrusion with >98% efficiency (50). Inside the protrusion (timepoint T_4_), only the scattering force F_s_ remains, enabling efficient optical trapping of the beads against the cavity wall and within the nanopore capture volume. There, target particles that are thermally released from the trapped beads can be detected rapidly and with single-molecule sensitivity without being whisked away by the flow or diffusing out of the capture volume. This nanopore-optofluidic integration increases the target local concentration inside the nanopore capture radius by many orders of magnitude. To quantify this effect, the local concentration enhancement can be calculated by dividing the target concentration at the nanopore by the bulk concentration. The former is defined as the number of targets trapped at the nanopore within the capture volume, and the latter is the concentration determined by the TACRE assay. The target number depends on both the number of trapped beads and the average number of RNAs on a bead, and it varies for each experiment. The values of the concentration enhancement factor for all of our tested samples are listed in *SI Appendix*, Tables S1 and S2 in supplementary material. The enhancement factor ranges from 8.3 × 10^3^ to 1.22 × 10^6^. This large increase is critical for implementing this high-throughput nanopore detection scheme with clinically relevant concentrations.

### Solid-Phase Extraction–Based Quantitative TACRE Assay.

A major advantage of our solid-state nanopore sensor platform in addition to being label-free and amplification-free is that only a few sample preparation steps are required to analyze complex biological sample matrices such as whole blood, plasma, urine, or nasal swabs. These include lysing of the clinical sample to expose the RNA targets and capturing them onto magnetic beads to ensure assay specificity.

**Fig. 2.**
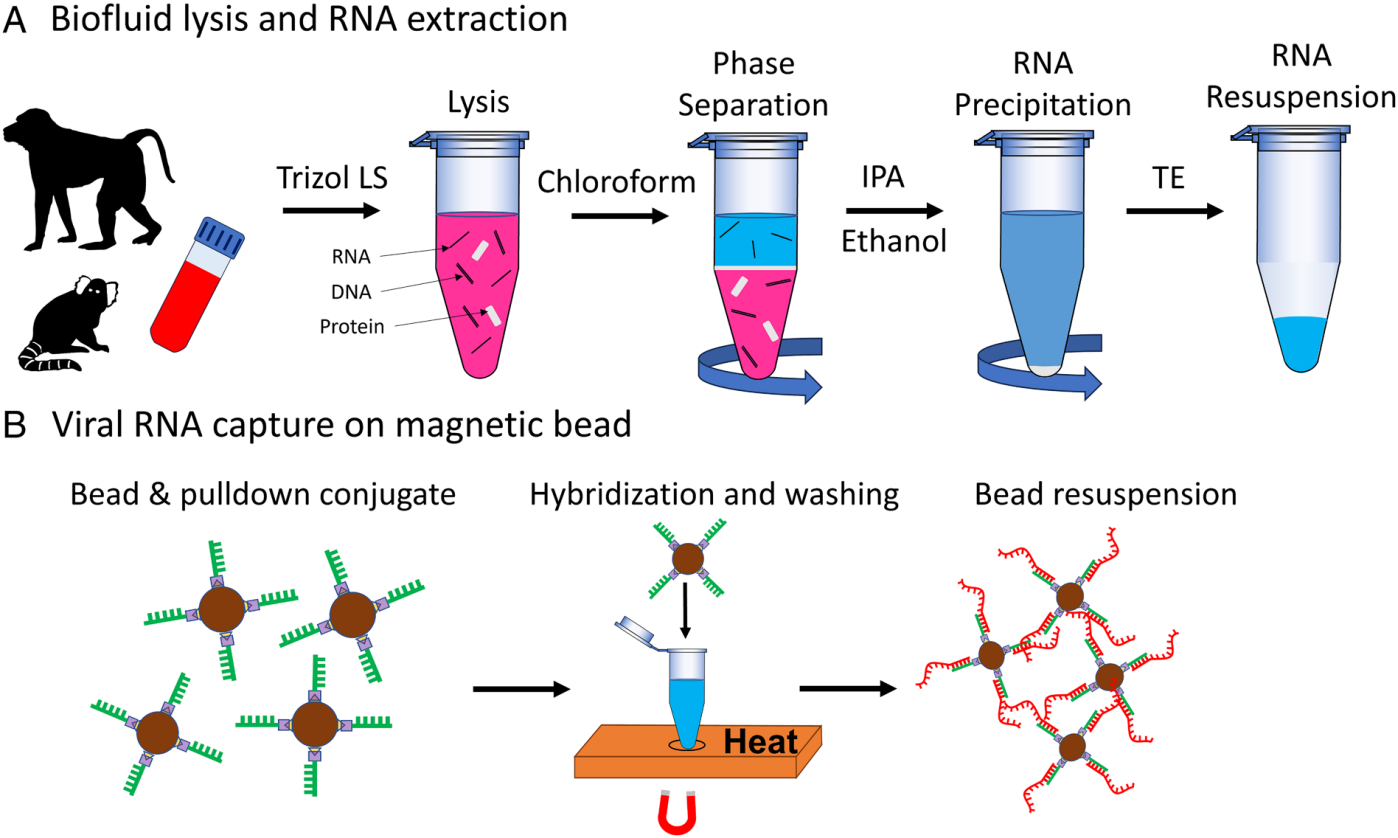
Biofluid lysis and RNA extraction. (*A*) Flowchart of the Trizol LS-based RNA extraction procedure. Typically, 250 μL of the sample volume is processed in a single cycle. The extracted RNA sample is resuspended in 30 to 50 μL of 1× TE buffer. (*B*) Viral RNA capture on magnetic microbeads. A 14 base pair long biotinylated pulldown oligonucleotide specific to Zika or SARS-CoV-2 viral RNA is attached with streptavidin-coated magnetic microbeads. Functionalized magnetic beads are mixed with extracted RNA sample for hybridization reaction. Only viral RNAs are captured by magnetically washing the sample. Finally, the immobilized magnetic beads are resuspended in nanopore working buffer solution.

We use a magnetic bead–based solid-phase extraction method for extracting specific viral RNAs from complex biofluids. This method enables concentrating target molecules on the surface of magnetic beads and removes unwanted constituents that could produce spurious nanopore signals. The sample is prepared in two steps: First, crude RNA is extracted from the lysed biofluid (Fig. 2*A*), and then, the magnetic bead–based solid-phase extraction assay is prepared for specific viral RNA detection (Fig. 2*B*). The details of these preparation steps are described in the *Materials and Methods* section. Briefly, 250 μL of the biofluid is lysed by Trizol LS, a phenol, guanidine isothiocyanate–based agent. RNAs are isolated from DNA and proteins by adding chloroform to the homogenized lysed sample. Finally, RNA is precipitated by adding isopropanol (IPA) to a smaller volume of TE buffer (50 to 30 μL). For quantitative analysis, the biofluid volume is tracked throughout the sample preparation steps. The RNA sample is then suspended in TE buffer. The solid-phase extraction process begins by functionalizing the magnetic bead surface with a target-specific pulldown sequence by a streptavidin–biotin-based attachment process (for details, see the *Materials and Methods* section). Each bead has roughly 240,000 pulldown binding sites available, offering a wide dynamic range for viral RNA concentration analysis. Next, a known number of functionalized beads (N_beads_) are mixed with the RNA sample until all targets are collected on the beads. A subset of these beads are optically trapped near the nanopore (N_trapped_), and the target RNAs are released and translocated. The cumulative number of translocations (N_xloc_) is equal to the total number of attached RNAs on the trapped beads. This provides an estimated number of viral RNAs attached per bead (*f*_*xloc:bead*_). The number of RNAs in the biofluid (N_RNA_) can then be determined by multiplying the total number of beads mixed with the RNA sample (N_beads_) with the average number of RNAs per bead estimated from the nanopore-based TACRE experiment (*f*_*xloc:bead*_)

[1] $$N_{\text{RNA}}=N_{\text{beads}}\;\times \;f_{\mathrm{xloc}:\mathrm{bead}},$$

where

[2] $$f_{\mathrm{xloc}:\mathrm{bead}}=\frac{N_{\text{xloc}}}{N_{\text{trapped}}}.$$

Finally, the concentration of the viral RNA from an unknown biofluid (*C_RNA_*) is calculated by dividing the estimated number of RNAs by the initial biofluid volume before lysis.

[3] $$C_{\mathrm{RNA}}=\frac{N_{\text{RNA}}}{V_{\text{biofluid}}}.$$

We note that the TACRE assay is a direct target detection method and thus quantitative and calibration-free, unlike qPCR. The choice of the number of functionalized beads mixed with the RNA sample is critical for obtaining lower LODs. A lower number of beads can target a lower number of RNAs but requires longer analysis time for collecting a sufficient number of trapped beads inside the nanopore capture radius. To keep the experimental analysis time within a few minutes for the lowest viral loads, the number of beads was chosen to result in approximately 1 to 100 targets per bead-based on the qPCR reference measurements. In the absence of a qPCR value, a viral load of 10^3^/mL was assumed. All assay and experimental information is listed in Tables 1 and 2 for Zika- and SARS-CoV-2-infected animals, respectively.

**Table 1.** Zika-infected marmoset biofluid sample preparation summary

Sample	Day 3	Day 9	Day 14
Blood	N_bead_ = 4,200 $f_{RNA:Bead}$ = 1.88 V_biofluid_: 250 μL $f_{xloc:bead}$ = 2.9 N_trapped_ = 21 N_xloc_ = 61	Not detected	N/A
Urine	Not detected	N_bead_ = 600 $f_{RNA:Bead}$ = 1.42 V_biofluid_: 500 μL $f_{xloc:bead}$ = 15.5 N_trapped_ = 2 N_xloc_ = 31	N/A
Semen	N/A	N_bead_ = 625 $f_{RNA:Bead}$ = 472 V_biofluid_: 62.5 μL $f_{xloc:bead}$ = 655 N_trapped_ = 4 N_xloc_ = 2,620	N_bead_ = 12,000 $f_{RNA:Bead}$ = 2.03 V_biofluid_: 250 μL $f_{xloc:bead}$ = 5.3 N_trapped_ = 10 N_xloc_ = 53

**Table 2.** SARS-CoV-2-infected baboon biofluid sample preparation summary

Sample	Day 2	Day 7	Day 10	Day 14	Day 18	Day 21
BAL	N_bead_ = 14,400 $f_{RNA:Bead}$ = 23.1 V_biofluid_: 250 μL $f_{xloc:bead}$ = 20.8 N_trapped_ = 5 N_xloc_ = 104	N/A	N/A	Not detected	N/A	Not detected
NPT	N_bead_ = 6,000 $f_{RNA:Bead}$ = 100 V_biofluid_: 8.75 μL $f_{xloc:bead}$ = 134.6 N_trapped_ = 10 N_xloc_ = 1,346	N_bead_ = 12,000 $f_{RNA:Bead}$ = 28 V_biofluid_: 250 μL $f_{xloc:bead}$ = 34.11 N_trapped_ = 18 N_xloc_ = 614	N_bead_ = 3,600 $f_{RNA:Bead}$ = 1.03 V_biofluid_: 600 μL $f_{xloc:bead}$ = 1 N_trapped_ = 3 N_xloc_ = 3	N_bead_ = 1,800 $f_{RNA:Bead}$ = ND V_biofluid_: 600 μL $f_{xloc:bead}$ = 22.63 N_trapped_ = 8 N_xloc_ = 181	N_bead_ = 1,800 $f_{RNA:Bead}$ = ND V_biofluid_: 600 μL $f_{xloc:bead}$ = 8.2 N_trapped_ = 5 N_xloc_ = 41	N_bead_ = 1,800 $f_{RNA:Bead}$ = 1.24 V_biofluid_: 600 μL $f_{xloc:bead}$ = 2.17 N_trapped_ = 6 N_xloc_ = 13
REC	Not detected	N_bead_ = 30,000 $f_{RNA:Bead}$ = 27 V_biofluid_: 250 μL $f_{xloc:bead}$ = 27.17 N_trapped_ = 6 N_xloc_ = 163	N_bead_ = 96,000 $f_{RNA:Bead}$ = 112 V_biofluid_: 250 μL $f_{xloc:bead}$ = 71.67 N_trapped_ = 9 N_xloc_ = 645	N_bead_ = 1,800 $f_{RNA:Bead}$ = 0.43 V_biofluid_: 250 μL $f_{xloc:bead}$ = 0 N_trapped_ = 2 N_xloc_ = 0	Not detected	Not detected

### Optofluidic-Nanopore Sensor Characterization for Quantitative Viral RNA Analysis.

**Fig. 3.**
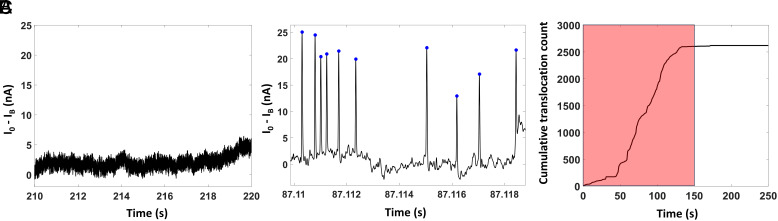
Trap-assisted capture rate enhancement experiment results with a semen sample from Zika-infected marmoset. The sample was collected on the 9th day after inoculation. (*A*) Baseline-subtracted nanopore ionic current shows no translocation signal without the presence of target RNA in the fluidic channel. (*B*) Characteristic translocation signals appear while the target RNA–conjugated magnetic beads are trapped under the nanopore and heated to 45 °C to release target RNAs from the beads. The blue dots indicate translocation signals detected by a custom-built peak detection algorithm. (*C*) Cumulative translocation count for the experiment with 4 trapped beads resulting in 655 translocations per bead (with 472 RNA per bead expected from PCR). The shaded red region indicates 2.5 min of heating window.

Each optofluidic nanopore chip was first characterized by filling it with 1× T50 buffer solution only. When an electrical voltage was applied between the nanopore and outlet reservoir, a steady baseline ionic current without spurious translocation signals (spikes) was established (Fig. 3*A*). We also verified that no false-positive signals were observed when empty (no targets) magnetic beads were optically trapped and heated under the nanopore.

We illustrate the typical process and analysis of the TACRE assay using the example of a marmoset semen sample and the PCR results as an independent reference (day 9, RT-qPCR viral load: 4.72 × 10^6^/mL). Here, 62.5 μL of the biofluid was processed, and an estimated average of 472 targets (*fRNA:bead*), based on the qPCR reference measurement, were attached to each magnetic bead. During the optical trapping stage, 4 target-carrying beads (N_trapped_) were trapped within the nanopore capture radius. The experiments were run with the highest voltage provided by the Digidata 1440A digitizer to maximize translocation rate and ionic current change, and characteristic translocation signals were observed when the simultaneous heat release and nanopore signal acquisition were started (Fig. 3*B*). While a smaller nanopore size would have further increased the relative current change ΔI/I, our experimental conditions produced translocation signals with very high signal-to-noise ratio, indicating that nanopore size did not limit our analytical sensitivity. In total, 2,620 translocation signals (N_xloc_) were observed, indicating the total number of released Zika viral RNAs from the trapped beads inside the nanopore capture volume. Statistical analysis of these translocation signals in a depth-duration scatter plot shows pronounced clustering of the events, indicating that the blockade signals were created by the same type of biomolecule (*SI Appendix*, Fig. S3). The calculated average number of viral RNA per bead (*f*_*xloc:bead*_) is 655, corresponding to a viral RNA concentration according to Eq. **3** of 6.28 × 10^6^/mL, close to the qPCR reference value. We point out that no blocking of the relatively large 20 nm nanopore by the target RNA molecules was observed.

### Longitudinal Viral Load Study with Virus-Infected Nonhuman Primates.

After characterizing the TACRE assay for Zika viral RNA quantification from the infected marmoset semen samples, we applied the assay in a comprehensive longitudinal study of two highly infectious diseases to track viral loads over the course of an infection for multiple biofluid samples.

**Fig. 4.**
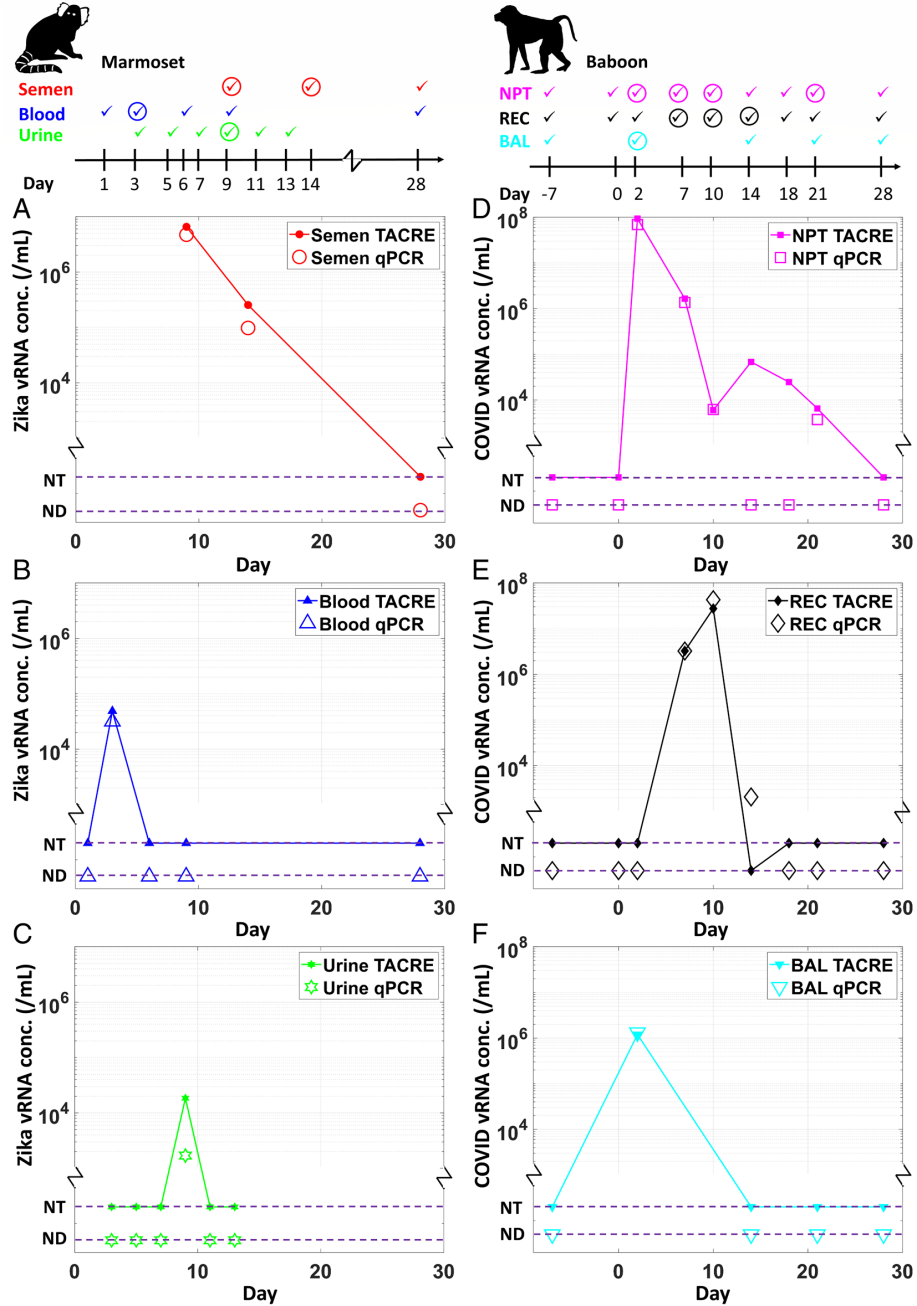
Longitudinal nanopore TACRE study of clinical biofluids. (*A*–*C*) Zika-infected marmoset and (*D*–*F*) SARS-CoV-2-infected baboon biofluid samples. Viral RNA loads were measured from six different types of clinical samples, i.e., semen, urine, whole blood, NPT swab, REC swab, and BAL. The *Top* panel shows the sample collection frequency along the longitudinal study. A checkmark indicates a day on which a sample was collected and tested with qPCR. A checkmark inside a circle indicates that the qPCR assay provided a result for the collected sample (solid marker: TACRE calculated value; open marker: qPCR obtained value). The dashed lines indicate that no viral load was detected (ND, PCR) or samples were not tested (NT, TACRE).

A 4-wk longitudinal study was designed with a Zika virus–infected marmoset as a nonhuman primate model. Three different biofluids (semen, urine, and whole blood) were collected from the animal at predetermined intervals. Details of the longitudinal study design and sample collection methods are provided in the *Materials and Methods* section. The collected samples were lysed, exposed to Zika virus–specific functionalized magnetic beads, and run through the optofluidic nanopore chip for label and amplification-free quantification. The results are summarized in Fig. 4 *A*–*C*. The *Top* panel shows the timeline of sample collection events (denoted by checkmarks) for the different fluids. Circles around the checkmark denote a positive RT-qPCR result, indicating that most PCR measurements were unsuccessful, likely due to very low viral loads or the complexity of the PCR process even when carried out in a highly specialized core lab.

Fig. 4 *A*–*C* compares the results of the qPCR (open symbols) and nanopore measurements (filled symbols) for all three sample types. The dashed lines denote samples for which PCR did not produce a result (ND) and, thus, were not tested with the nanopore assay (NT). We first note that the nanopore TACRE assay yielded results for all clinical sample types and correctly reproduces the qualitative trends observed with qPCR. Specifically, semen emerged as the most suitable sample type, showing the highest viral loads and largest observable period, in agreement with previous studies (51). While urine and blood samples produced only limited viral load values for both assay types, we were able to conclude from the nanopore assay that the viral load peaks earlier in the infection, and that urine can be used as a more readily available sample fluid if semen is too difficult or impossible to obtain. Importantly, while comparable, the concentration values obtained by the nanopore sensor were consistently higher than the qPCR results. We attribute this to the fact that our assay is simpler and more direct while PCR has numerous steps (reverse transcription, amplification, and standard curve calibration) that can lead to target loss and errors.

Another 4-wk longitudinal study was performed with a SARS-CoV-2-infected baboon. This animal model was developed early in the pandemic with the goal of determining the suitability of different fluids for diagnostics and assistance with vaccine development. Here, three types of biofluids NPT, REC swab, and BAL) were collected, processed, and analyzed with the same protocols as the Zika samples. Our results follow the same trends as in the Zika study, thus validating the versatility of the nanopore sensor approach. Again, the timeline on top of Fig. 4 *D*–*F* shows that less than 50% of the collected samples produced a positive PCR result. As was the case for the Zika study, the nanopore assay successfully detected and quantified viral RNAs from all types of biofluids (Fig. 4 *D*–*F*). This study showed that NPT and REC swab samples produced the highest viral loads, with the NPT sample peaking earlier and producing results over the longest time period. The temporally delayed response in the REC swab samples (Fig. 4*E*) indicates that both respiratory and gastrointestinal infections are caused by the SARS-CoV-2 virus as predicted recently (52). The viral load in the BAL samples followed the same course as in NPT samples but at ~100× lower concentrations, as observed both with PCR and the nanopore sensor (Fig. 4*F*).

Due to the small amounts of sample volume, we were not able to consistently run triplicate measurements. However, the validity of our measurements was borne out by the good agreement with the PCR reference values across all samples and days. Indeed, we find very consistent results for both qPCR and the nanopore sensor assays across all virus and sample types. Our label- and amplification-free analysis method produced results for all PCR-positive samples with the exception of a single SARS-CoV-2 sample (day 14, REC swab), in which case the PCR concentration was very low and very little sample volume was available for the nanopore measurement. On the other hand, we ran two of the samples for which PCR did not produce a result and that represented a gap in the longitudinal study (SARS-CoV-2, NPT, days 14 and 18). As can be seen in Fig. 4*D*, we were able to obtain a quantitative viral load value for both samples that is highly consistent with the dynamic trend that this fluid exhibited over time. Since the TACRE assay does not have a fundamentally lower LOD than PCR, we did not test samples at the beginning and end of the infection when viral loads are extremely low. We thus conclude that our approach performs at least as well as RT-qPCR, but with significantly reduced experimental complexity.

## Discussion

We have demonstrated an integrated optofluidic-nanopore platform for label-free and amplification-free quantification of viral RNA from clinical biofluid samples. A modified solid-phase extraction method was utilized to provide selectivity and easy target isolation by immobilizing target viral RNAs on the microbead surface out of a complex matrix. Optical trapping–assisted microbead delivery and thermal release of target particles near the nanopore sensor enabled rapid, high-throughput diagnosis by increasing the local target concentration up to over one million times. This TACRE nanopore assay was utilized for quantitative viral RNA analysis from infected animal biofluid samples, showing a performance comparable to, and sometimes exceeding, that of the more complex, nucleic acid amplification-based RT-qPCR gold standard method. Incorporation of this optofluidic-nanopore platform in a longitudinal viral load monitoring study comprising two lethal viral infections (i.e., Zika and SARS-CoV-2) and six different types of biofluids showed the versatility of this assay, paving a unique way toward solid-state nanopore-based molecular diagnosis from clinical samples without the need of nucleic acid sequencing and amplification. In the future, the sample preparation steps for the assay can be further simplified and miniaturized. A lightweight, portable separation disk can be utilized to extract the total RNA (53). Moreover, on-chip automatic sample preparation and delivery steps can be implemented to lower the required sample volume (54, 55). Furthermore, the approach can be extended to multiplexed analysis using various strategies. These include sequentially exposing the sample to differently functionalized beads and delivering these groups of beads to one or more nanopores, defining multiple microfluidic channels with nanopores working in parallel and by routing beads with different target biomarkers to different channels, or simultaneous detection with a single nanopore by designing pulldown sequences for different melting temperatures and then detecting the target molecules in order of increasing with the lowest pulldown melting temperature.

In summary, this work points the way toward a class of universal integrated nanopore sensors that combine high performance with low to medium complexity. These devices can find applications as POC diagnostics, research tools for assisting in the development of animal models, and many other fields.

## Materials and Methods

### Nanopore-Integrated Optofluidic Device Fabrication.

The nanopore-integrated optofluidic devices were fabricated utilizing a silicon nanofabrication process on a flat 100 mm diameter silicon wafer (50), and a side view of the cross-section of the device is shown in *SI Appendix*, Fig. S1*A*. First, a 3 mm long optofluidic channel structure was defined by anisotropically etching the silicon substrate based on a predesigned mask in an STS ICP Multiplex ASE reactive ion etch (RIE) tool. The microfluidic channel mask was composed of multichannel inlets and outlets for particle delivery, a 100 µm long optofluidic region for light–matter interaction, and a 20 µm long protrusion cavity for isolating optically trapped particles from the fluid flow. The etched channel was 10 µm high and 14 μm wide. A second etch using the RIE provided a pedestal for intersecting solid-core (SC) waveguides, which are meant to be 3 µm lower than the top of the liquid channel so the optical mode profile of the waveguides aligns with the center of the optofluidic channel. This second etch also defines the liquid channel wall thickness to approximately 2.5 µm. The channel wall was then thermally converted from silicon to silicon dioxide (refractive index n = 1.44) in a furnace at 1,100 °C for 10.5 h. Silicon dioxide grown outside the channel wall provided the bottom, low-index cladding for the SC waveguides. A 3 μm thick, high-index (n = 1.51) plasma-enhanced chemical vapor deposition (PECVD) silicon dioxide layer was then deposited over the wafer. A 10 µm wide waveguide was patterned by photolithography and RIE to form the core of the SC waveguide intersecting the optofluidic channel. A 300 nm thin PECVD silicon dioxide membrane was grown to cover the microfluidic channel by first filling the channel with a sacrificial polymer (SU8-2000.5) through capillary action. The naturally formed meniscus of the polymer supports and shapes the thin membrane (56). In order to provide mechanical strength to the membrane and cladding to the SC waveguide, a 2 μm thick low index (n = 1.44) silicon dioxide was deposited over the channel excluding the central trapping region (Fig. 1*B*). The sacrificial polymer was finally removed by chemical etching using sulfuric acid and hydrogen peroxide to create a hollow channel with a 300 nm thin suspended silicon dioxide membrane in the optofluidic region, paving an easier way to integrate a solid-state nanopore sensor to the optofluidic device. An electronic voltage-current measurement was performed to confirm the intactness of the 300 nm thin oxide membrane before the nanopore milling process (*SI Appendix*, Fig. S4).

An FEI Quanta 3D FEG Dualbeam with the gallium ion beam operating at 30 kV and 10 pA was used to mill a nanopore into the 300 nm thick silicon dioxide membrane on top of the protrusion cavity. To fabricate the nanopore, a 2 μm diameter circular microwell is first milled into the membrane to create a locally thinner membrane. A ~20 nm nanopore is then drilled through the remaining thinner oxide with 14~20 ms dwell time at a single point using Nanometer Pattern Generation System software (JC Nabity Lithography Systems). An additional tetraeythlorthosilicate-based nanopore shrinking step is added if the initial nanopore size exceeds the desired value or to patch any visible crack in the thinner oxide membrane (*SI Appendix*, Fig. S5). Both the microwell and nanopore fabrication processes are monitored by a scanning electron beam operating at 5 kV and 6.7 pA.

### Optical and Electronic Setup.

For optical trapping of magnetic microbeads, a 532 nm wavelength fiber laser (MPB Communications Inc.) was used. Single-mode fiber-coupled laser light was butt coupled to the SC waveguide. The microbead flow was monitored using a CCD camera (Andor Luca R, Oxford Instruments) connected with a custom-built microscope. A 50× long working distance objective lens (Olympus, SLMPlan, 0.45 NA, 15 mm WD) was used to illuminate and collect light from the device, and a 40 nm bandpass filter centered at 670 nm wavelength (Omega Optical LLC. 670DF40) was used to reject the laser light.

To enable thermal target release from the trapped magnetic microbeads, a Peltier heater (TES1 12703, Hebei I.T. (Shanghai) Co., Ltd.), connected to a temperature controller (LDC 3724B, ILX Lightwave), was employed. A 10 kΩ thermistor, acting as a feedback sensor, was placed on the heater surface and linked to the controller circuit to execute the proportional, integral, and derivative algorithm.

### Nanopore Signal Acquisition and Translocation Analysis.

A pair of Ag/AgCl electrodes were used to introduce electrical voltage between the microfluidic channel (cis) and the nanopore reservoir (trans). All reservoirs and the microfluidic channel were filled with 1× T50 buffer (50 mM NaCl, 10 mM Tris-HCl, filtered with 20 nm filter). The nanopore ionic current signal was amplified using a sensitive current amplifier (Axopatch 200B, Molecular Device), and a digitizer (Digidata 1440A, Molecular Device) was used to record the low pass filtered (cutoff frequency of 10 kHz) signal at a rate of 250 kSa/s. The recorded current trace was subsequently analyzed using a custom MATLAB program designed to identify translocation events. Briefly, the program first analyzes the noise characteristics of the baseline current signal by calculating its mean and SD. A user-defined detection threshold is set at 5 to 7× of the SD of the baseline ionic current. A reference trace is created to follow the trend of the baseline current signal. In each processing cycle, a group of five data points is considered together to down sample the signal, and the mean value of the group is treated as the new data point. This new data point is compared to the previously calculated reference data point, and if the difference does not exceed the threshold, the reference point is updated using a proportional control algorithm. Once the threshold is crossed, the program flags the event as a potential translocation event and calculates the peak height. If the current subsequently falls back below a predefined threshold within a user-defined time (in this case, 5× the expected dwell time), the program records the translocation dwell time, confirming the event as a genuine translocation. However, if the threshold is crossed due to a random baseline shift event, the ionic current amplitude remains above the predefined threshold value for the entire user-defined time window. In this case, the program discards the event as a timeout event.

### Ethics Statement.

All experimental procedures with animal samples were approved by the Institutional Animal Care and Use Committee of the Texas Biomedical Research Institute (IACUC, #1714 PC and #1528 CJ 10) and the University of California Santa Cruz (IACUC, #Schmh2104 and #Schmh 2207dn).

### Marmoset and Baboon Inoculation and Sample Collection.

A male marmoset (*Callithrix jacchus*) was inoculated with Zika virus, and three different bodily fluids (urine, whole blood, and semen) were sampled and serially monitored. The inoculation and sample collection method was described previously in ref. 57. The urine sample was collected on days 3, 5, 7, 9, 11, and 13 (considering the date of viral inoculation as day 0). The whole blood sample was collected on days 1, 3, 6, 9, and 28. The semen sample was collected on days 9, 14, and 28.

Similarly, a baboon (*Papio hamadryas anubis*) was inoculated with SARS-CoV-2 virus, and three different bodily fluids (BAL, nasopharyngeal throat swab, and REC swab) were sampled and serially monitored as described in ref. 58. The BAL was collected on days −7, 2, 14, 21, and 28. The NPT and REC swab sample were collected on days −7, 0, 2, 7, 10, 14, 18, 21, and 28.

### Total RNA Extraction from Animal Biofluids.

RNA extraction from the collected animal biofluids was performed in a biosafety level-2 cabinet using a Sorvall Legend Micro 21R Centrifuge (Thermo Scientific). All samples were inactivated by mixing with 750 μL of TRizol LS (250 μL sample + 750 μL of Trizol LS) maintaining a ratio of 3:1 between the volume of Trizol LS and the sample. If the volume of the sample was less than 250 μL, then 1× PBS was added to bring the volume of the sample to 250 μL. The Trizol LS (Invitrogen) protocol was then followed to extract total RNA for the downstream magnetic bead–based solid-phase extraction process. Briefly, 200 μL of chloroform was added and mixed to the lysed sample tube by pipetting up and down. The tube was then centrifuged for 15 min at 12,000 × g at 4 °C followed by a 3 min incubation period at room temperature. The clear upper aqueous layer (approximately 600 μL), which contains RNA, was carefully transferred to a new 1.5 mL tube. To precipitate RNA, 500 μL of IPA was added to the transferred liquid and mixed. The sample was then centrifuged for 10 min at 12,000 × g at 4 °C followed by a 10 min incubation period at room temperature. The supernatant was discarded, and the remaining pellet was resuspended by vortex in 1 mL of 75% ethanol. The sample was again centrifuged for 5 min at 7,500 × g at 4 °C. Extra ethanol was discarded and the RNA pellet was air-dried for 20 min. The isolated total RNA was eluted by 30 to 50 μL of nuclease-free water (Invitrogen) or 1× TE buffer (10 mM Tris and 0.1 mM ethylenediamine tetraacetic acid) by flicking the tube. Finally, the RNA solution was incubated in a water bath set at 55 to 60 °C for 15 min.

### Viral RNA Quantification Using qRT-PCR.

For independent viral load reference measurements, total ZIKV RNA was isolated from bodily fluids (semen, saliva, and urine) using TRIzol LS reagent (Invitrogen) according to the manufacturer’s instructions. Ten micrograms of yeast RNA as a carrier and 30 μg of GlycoBlue Coprecipitant (Invitrogen) were added during the extraction procedure. The RNA pellet was resuspended in a volume of 50 μL nuclease-free water, and 5 μL of RNA was used to quantify the viral titer. Quantitative RT-PCR was performed using the RNA Ultrasense One-Step RT-PCR system (ThermoFisher) on an Applied Biosystems 7500 Real-Time instrument at 40 °C for 30 min, followed by denaturation at 95 °C for 10 min and then thermocycling for 40 cycles at 95 °C for 15 s and 60 °C for 1 min. The sequence of the ZIKV primers used is forward 5′-AAR TAC ACA TAC CAR AAC AAA GTG-3′, reverse 5′-TCC RCT CCC YCT YTG GTC TTG-3′, probe 5′-/56-FAM/CTY AGA CCA /ZEN/GCT GAA R/3IABkFQ/-3′ (Integrated DNA Technologies) (59).

SARS-CoV-2 viral RNA was quantified via qRT-PCR, as previously described (58, 60). The CDC-developed 2019-nCoV_N1 assay was used to target a region of the N gene. Briefly, samples were inactivated using TRIzol LS Isolation Reagent (Invitrogen), and RNA was extracted using the EpMotion M5073c Liquid Handler (Eppendorf) and the NucleoMag Pathogen kit (Macherey-Nagel). MS2 phage (*Escherichia coli* bacteriophage MS2, ATCC) is spiked in as an internal efficiency control. The TaqPath 1-Step RT-qPCR Master Mix (Life Technologies) was used for qRT-PCR, using 5 µL of the extracted RNA material. Assays were performed on a QuantStudio 3 instrument (Applied Biosystems) with the following cycling parameters: Hold stages, 2 min at 25 °C, 15 min at 50 °C, and 2 min at 95 °C. PCR stages: 45 cycles of 3 s at 95 °C, 30 s at 60 °C. Primer and probe info: 2019-nCoV_N1-F: GACCCCAAAATCAGCGAAAT (500 nM); 2019-nCoV_N1-R: TCTGGTTACTGCCAGTTGAATCTG (500 nM); 2019-nCoV_N1-P FAM/MGB probe: ACCCCGCATTACGTTTGGTGGACC (125 nM).

### TACRE-Based Viral RNA Quantification Assay Component.

A magnetic bead–based solid-phase extraction method was utilized to capture specific gene of the viral RNA sequences from the total RNA sample. Streptavidin-coated magnetic microbeads of 1 μm diameter (4 mg/mL) were purchased from New England Biolabs. The beads bind with 500 pmol of single-stranded 25 bp biotinylated oligonucleotide per mg.

Two 14 base pair long biotinylated ssDNA pulldown sequences (Zika: 5′-/5BiotinTEG/GTTTTGGTATGTGT-3′ and SARS-CoV-2: 5′-/5BiotinTEG/CATTTCGCTGATTT -3′) were purchased from Integrated DNA Technologies (IDT). The melting temperature of both pulldown sequences in 50 mM Na+ salt is 35.1 °C (http://biotools.nubic.northwestern.edu/OligoCalc.html). The pulldown sequence for Zika viral RNA quantification experiment was designed to complement a part of NS5 region of the viral genome (nt. 9,275 to 9,288; Zika virus strain ZikaSPH2015, complete genome, NCBI Reference Sequence: KU321639), and the pulldown sequence for SARS-CoV-2 experiment was designed to complement a part of the ORF1ab region of the viral genome (nt. 28,294-28,307; Severe acute respiratory syndrome coronavirus 2 isolate Wuhan-Hu-1, complete genome, NCBI Reference Sequence: NC_045512.2)

For pulldown bound magnetic bead preparation, 5 μL of the stock magnetic bead (2 × 10^7^ beads) was magnetically washed and resuspended in 20 nm syringe filtered 1× T50 buffer solution. The washed magnetic beads were then added to biotinylated synthetic capture pulldown oligomers so that the ratio between the number of available binding sites and the number of pulldown oligomers is 1:6. The sample was well mixed in a rotary mixture for 1 h, and the unbound excess pulldown sequences were discarded by magnetic wash. Afterward, the pulldown functionalized beads were resuspended at a suitable concentration (10^7^ to 10^5^ beads/mL) for downstream sample preparation.

The viral RNAs were immobilized on the pulldown functionalized magnetic beads by a nucleic acid hybridization process. Usually, RNA was extracted from initial biofluid of volume 250 μL and resuspended into 50 μL solution. The sample was first heated at 95 °C for 5 min to linearize the secondary structure, and then 6 μL of 10^6^/mL concentration pulldown functionalized beads were mixed for sequence specifically capturing viral RNAs. The beads functionalized with pulldown sequences targeting SARS-CoV-2 virus were mixed with infected baboon biofluid-derived RNA samples, and similarly, Zika virus targeting pulldown functionalized beads were mixed with infected marmoset biofluid-derived RNA samples. The initial biofluid volume and the number of pulldown functionalized beads were carefully chosen for each biofluid sample to obtain the optimum experimental condition assuring a LOD comparable to qPCR. The mixed sample was incubated at 30 °C water bath for 2 min to facilitate the annealing process. To further increase the viral RNA capture process, the mixed sample was heated at 95 °C for 1 min and annealed at 30 °C for 2 min. This offers an extended target capture volume for a single magnetic bead ensuring higher efficiency. After five capturing and annealing cycles, the target connected bead sample was incubated in an ice bath for 30 min. Finally, the unattached RNAs were carefully discarded by magnetic wash, and the viral RNA-connected magnetic beads were suspended in nanopore working buffer (1× T50) with 0.5% Tween 20 surfactant for lossless bead delivery through the microfluidic channel.

For a single viral RNA quantification experiment, 6 μL of target functionalized beads with chosen concentration was added to the inlet reservoir. The optical trapping experiment was run for 10 to 20 min. The heater was turned on after the optical trapping experiment and set at 45 °C (~10 °C higher than the melting temperature of the pulldown sequence) for 2.5 min.

The carboxylate-modified fluorescent microbeads (1 μm diameter) for characterizing the optical trapping performance of the experimental devices were purchased from ThermoFisher Scientific (Excitation/Emission: 625/645 nm). The beads were subjected to brief vortex and sonication and diluted to 10^7^ to 10^6^/mL concentration in 1× T50 buffer solution. Tween 20 surfactants were added to the bead solution at 0.5% final concentration for avoiding bead aggregation and nonspecific bead binding events to the microfluidic channel surface. Each characterization experiment takes 6 μL of the fluorescent bead sample.

## Supplementary Material

Appendix 01 (PDF)

## Data Availability

All study data are included in the article and/or *SI Appendix*.
