# Supplementary material for: Label-free and amplification-free viral RNA quantification from primate biofluids using a trapping-assisted optofluidic nanopore platform
Source: Proc Natl Acad Sci U S A. 2024 Apr 10;121(16):e2400203121. doi: 10.1073/pnas.2400203121 (PMC11032468; doi:10.1073/pnas.2400203121)
Supplement: Supplementary file 1 — Appendix 01 (PDF) [file pnas.2400203121.sapp.pdf]

PNAS

Supporting Information for

Label-free and amplification-free viral RNA quantification from primate biofluids using a trapping-assisted optofluidic nanopore platform

Mohammad Julker Neyen Sampad[a*], S M Saiduzzaman[a], Zach J. Walker[b], Tanner N. Wells[b], Jesse X. Wayment[b], Ephraim M. Ong[b], Stephanie D. Mdaki[c], Manasi A. Tamhankar[c], Thomas D. Yuzvinsky[a], Jean L. Patterson[c], Aaron R. Hawkins[b], and Holger Schmidt[a]

[a]School of Engineering, University of California, Santa Cruz, CA 95064, USA; [b]Electrical and Computer Engineering Department, Brigham Young University, Provo, UT 84602, USA; [c]Texas Biomedical Research Institute, San Antonio, TX 78227, USA.

*To whom correspondence may be addressed: Mohammad Julker Neyen Sampad.

Email: msampad@ucsc.edu

This PDF file includes:

Figures S1 to S5
Tables S1 to S2
SI References

a)

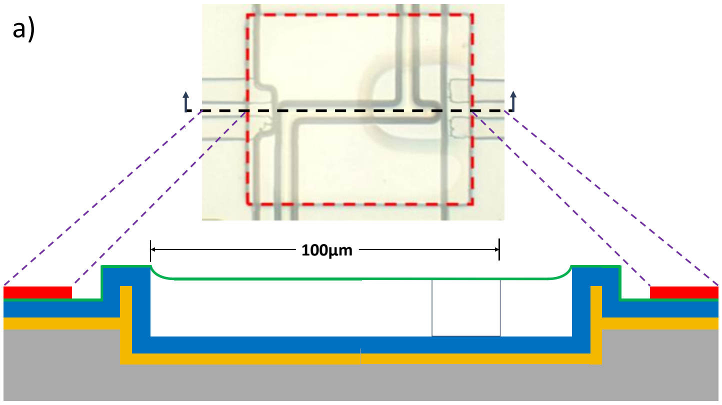

b)

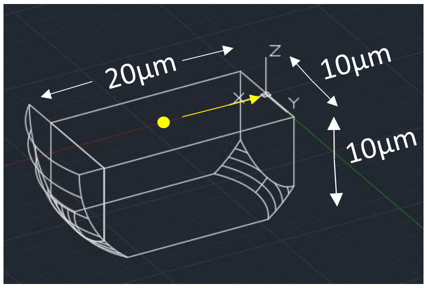

Fig. S1. a, Cross-sectional view of the optofluidic region. From left, a vertically aligned solid-core ridge waveguide made with high refractive index PECVD silicon dioxide (in blue, t = 3µm, n = 1.51) brings light into 100µm long horizontal part of the microfluidic channel. At the intersection, a channel wall defining thin layer of silicon is thermally converted to silicon dioxide (in yellow, t = 2µm, n = 1.44) for low-loss optical transmission. The channel is selectively covered by 300nm thin suspended silicon dioxide nanomembrane (in green) inside the red dashed region, where the rest of the area has an additional thicker low refractive index silicon dioxide layer (in red, t = 2µm, n = 1.44) acting as cladding layer for the waveguide as well as for providing mechanical protection to the chip. At the right end of the optofluidic region there is a protrusion, where the optically trapped beads are isolated and a nanopore sensor is integrated for rapid detection. Grey layer indicates silicon substrate. **b,** Nanopore capture volume calculation. The cross-section of the protrusion in the microchannel is 10µm height x 10µm width x 20µm length. The nanopore is located at the center of the top surface of the protrusion (yellow dot). The intersection between the semi-sphere with a radius of 13.4 µm, which is capture radius of similar size nanopore (~20 nm diameter) found in (1) and the microfluidic channel is drawn in AutoCAD. The volume of this region is 2,187 µm^3 or 2.187x10^{-9} mL, and represents the capture volume of the nanopore for the integrated optofluidic nanopore chip.

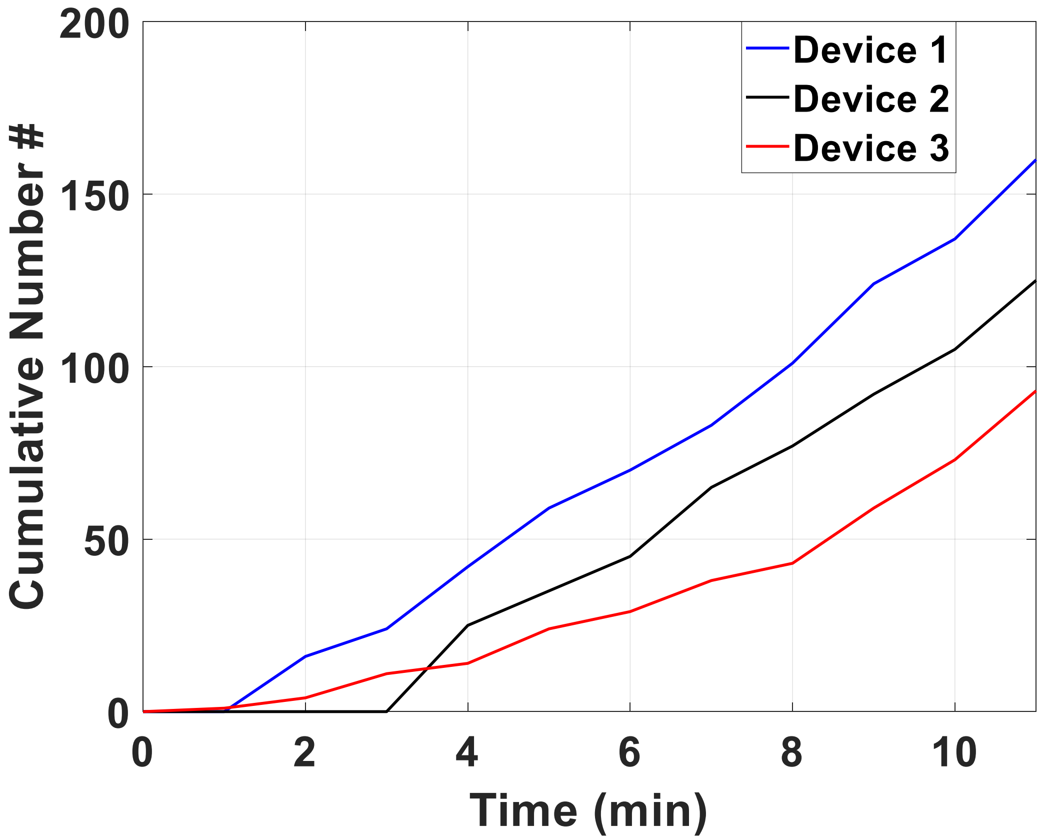

Fig. S2. Natural evaporation controlled microbead delivery for optical trapping. Cumulative number of trapped microbeads vs experiment time shows bead delivery characteristics in for three different optofluidic-nanopore devices. This experiment was done with 1um diameter fluorescent beads at $4x10^7$/mL concentration suspended in 1xT50 (50mM NaCl, 10mM Tris-HCl) buffer with 0.5% Tween 20 surfactant. The inlet reservoir was filled with 6μL of bead solution preceded by a drop (~1μL) of mineral oil and the outlet reservoir was filled with 6.7μL of 1xT50 buffer only. The cumulative trapped bead value varies among three different devices due to slight differences in natural evaporation rate or sample handling.

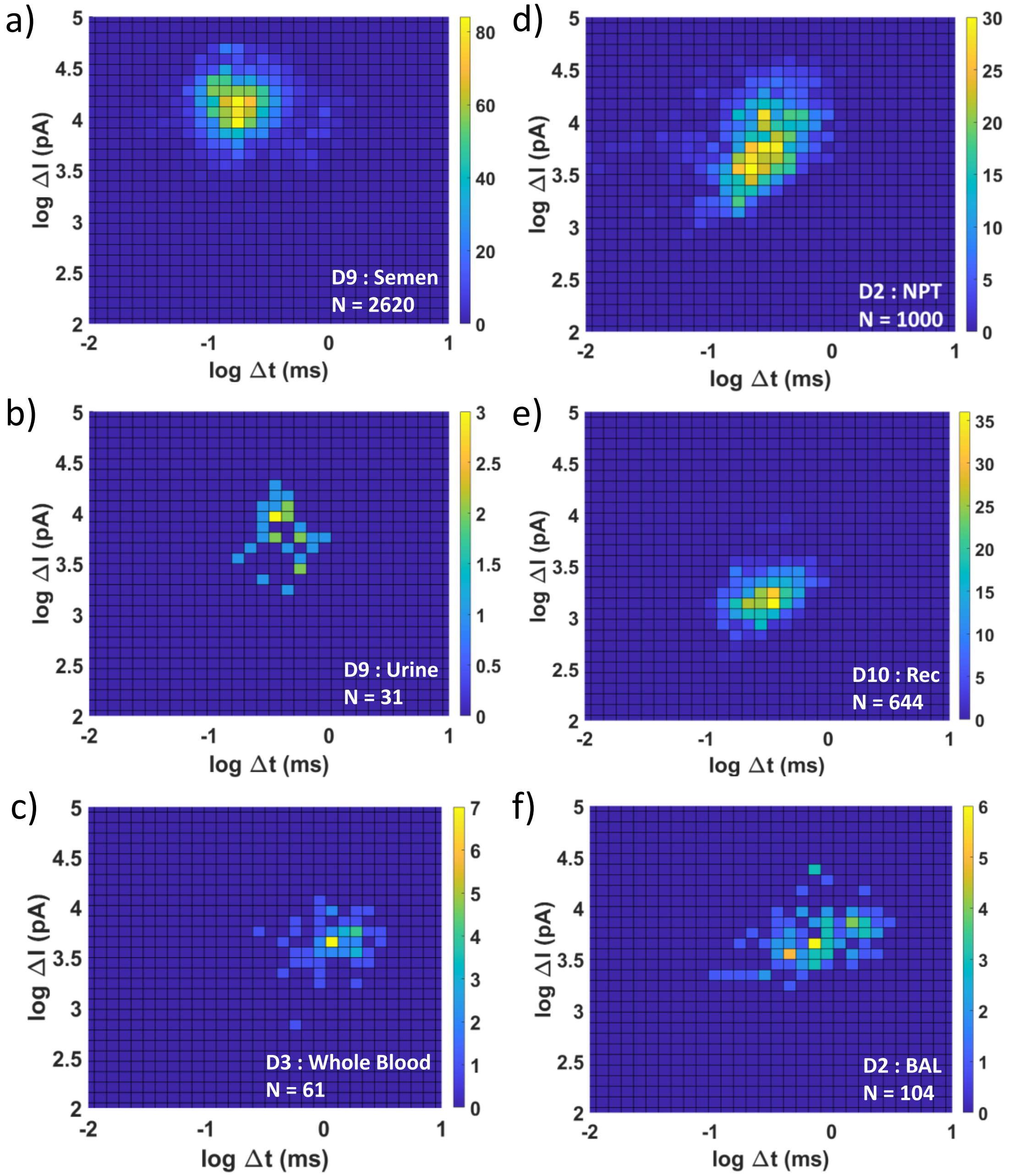

Fig. S3. Statistical analysis of translocation signals for TACRE experiments with six different biofluids. Biofluid type and collected date is indicated. As expected, all the scatter-heat plots show a well-defined cluster, indicating a single type of particle translocation obtained from all six different biofluids.

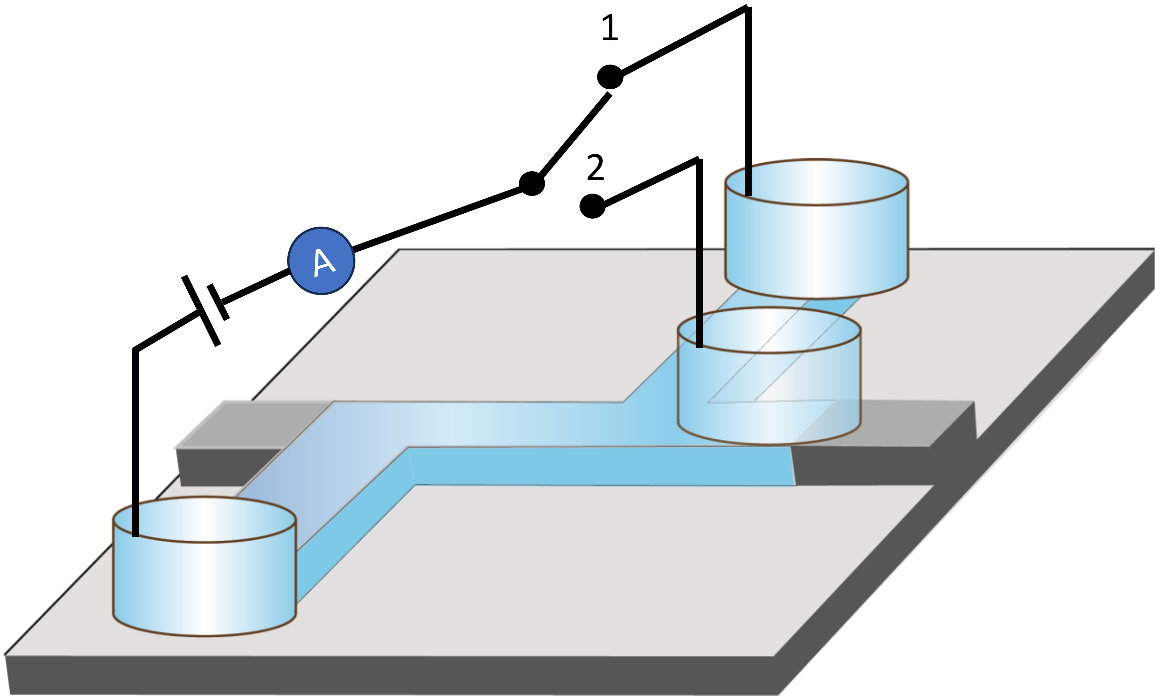

Fig. S4. Experimental setup for electronic voltage-current based 300nm thin membrane intactness test. The inlet and outlet of the microfluidic channel are accessed by wax mounted reservoirs and a third reservoir is mounted on the central optofluidic region where the membrane thickness is 300nm. The reservoirs and the channel is filled with 1xT50 (50mM NaCl, 10mM Tris-HCl) salt solution. With a DC voltage application system and Ag/AgCl electrode pair, electrical current along the microfluidic channel was observed when the SPDT switch was at position #1. If the switch was moved to position #2, leakage current across the 300nm thin membrane was recorded. For an intact membrane device before nanopore milling, a non-zero ionic current between the inlet-outlet reservoirs and a zero current between the inlet-central reservoirs is expected.

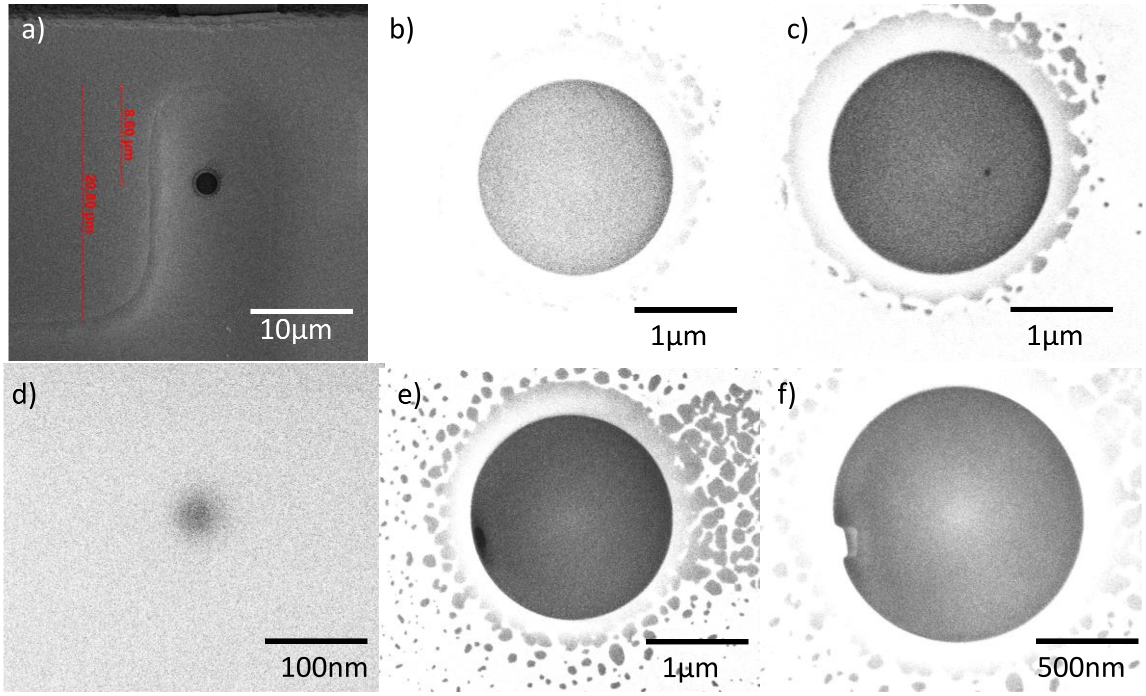

Fig. S5. SEM micrographs of nanopore fabrication steps on top of the protrusion cavity. **a,** Top-down view of a 2μm diameter microwell made by milling with focused ion beam. The microwell is located at the center of the channel's top surface and 8.6μm away from the protrusion wall. **b,** Zoomed in view of the thinned oxide membrane in the microwell. The microwell was formed by focused ion beam (FIB) at 30kV, 10pA and patterned for 8~9 minutes. **c,** Top-down view of the nanopore milled in the membrane. NPGS (Nanopattern Generation System) parameter: FIB - 30kV, 10pA, single dose, dwell time: 14~20ms. **d,** zoomed in view of the milled nanopore. Nanopore diameter: 20nm. **e,** if a visible crack at the corner of microwell appears due to non-uniformity of the membrane thickness, the microwell fabrication was stopped immediately at this point. **f,** no visible crack in the microwell membrane after the TEOS based silicon dioxide patching.

Table S1. Local target concentration enhancement factor for TACRE experiments with Zika infected Marmoset samples.

**Sample**	**Day 3**	**Day 9**	**Day 14**
Blood	5.72×10^5	Not Detected	N/A
Urine	Not Detected	7.66×10^5	N/A
Semen	N/A	3.053×10^5	9.54×10^4

Table S2. Local target concentration enhancement factor for TACRE experiments with SARS-CoV-2 infected Baboon samples.

Sample	Day 2	Day 7	Day 10	Day 14	Day 18	Day 21
BAL	$3.96x10^4$	N/A	N/A	Not Detected	N/A	Not Detected
NPT	$8.295x10^3$	$1.7x10^5$	$2.29x10^5$	$1.22x10^6$	$7.62x10^5$	$9.145x10^5$
REC	Not Detected	$1.35x10^4$	$1.07x10^4$	Not Detected	Not Detected	Not Detected

SI References

1. M. J. N. Sampad, *et al.*, Optical trapping assisted label-free and amplification-free detection of SARS-CoV-2 RNAs with an optofluidic nanopore sensor. *Biosens. Bioelectron.* **194**, 113588 (2021).
